# Gravity and magnetic anomalies of earthquake-prone areas in the southwestern Ulleung basin margin, East Sea (Sea of Japan)

**DOI:** 10.1038/s41598-022-21462-3

**Published:** 2022-10-13

**Authors:** Chang Hwan Kim, Kwang-Hee Kim, Soon Young Choi, Won Hyuck Kim, Hyun Ok Choi, Chan Hong Park

**Affiliations:** 1 Dokdo Research Center, East Sea Research Institute, Korea Institute of Ocean Science & Technology, Uljin, Republic of Korea; 2 Department of Geological Science, Pusan National University, Busan, Republic of Korea

**Keywords:** Solid Earth sciences, Geophysics

## Abstract

Submarine earthquakes have increased in the southwestern Ulleung Basin adjacent to the Korean Peninsula. This study analyzed the gravitational and magnetic properties of the three earthquake-prone areas (Hupo Bank and offshore regions near Pohang and Ulsan) in the basin. The basin was affected by tensile and compressive stresses during the formation of the East Sea. The southern Hupo Bank and the Pohang offshore exhibited high gravity anomalies and strong magnetic anomalies. Hupo Bank was separated from the peninsula and earthquakes in this region have been influenced by crustal fractures that facilitated igneous activities during the formation of the basin. Dense volcanic rocks and seaward dipping reflectors along the Pohang coast and continental slope suggest magmatic activities during the formation of the East Sea. Comparatively, the Ulsan offshore, with a thick sedimentary layer, exhibited a slightly higher gravity anomaly than the surrounding area, but no significant differences in the magnetic anomaly. Sequential tensile and compressive stresses related to the creation of the basin produced complex tectonic structures in this region. The magnetic tilt derivative results suggest that earthquakes were located near magnetic source boundaries. The results show that it is important to monitor earthquake-prone areas with gravity and magnetic anomalies.

## Introduction

Earthquakes can cause major disasters but are difficult to predict. In particular, land earthquakes threaten human life by destroying structures such as buildings and bridges. Submarine earthquakes also occur, many of which have recently caused crustal displacement, tsunamis, and the destruction of coastal areas. After the 2011 Tohoku earthquake occurred off the Pacific coast of Japan, earthquakes have also been observed in the southeastern Korean Peninsula and the southwestern part of the Ulleung Basin^1–5^. More recently, the Gyeongju and Pohang earthquakes in Korea, which occurred on Sep. 12, 2016 and Nov. 15, 2017, respectively, caused damage to the value of more than 100 billion won^1,2,4,6,7^. In May 1983 and Jul. 1993, tsunamis resulting from earthquakes offshore of Akita and Hokkaido, along the western coast of Japan, caused substantial damage (3 people (dead and missing), 79 damaged houses, 81 damaged fishing boats) to the east coast of the Korean Peninsula^8,9^.

**Figure 1:**
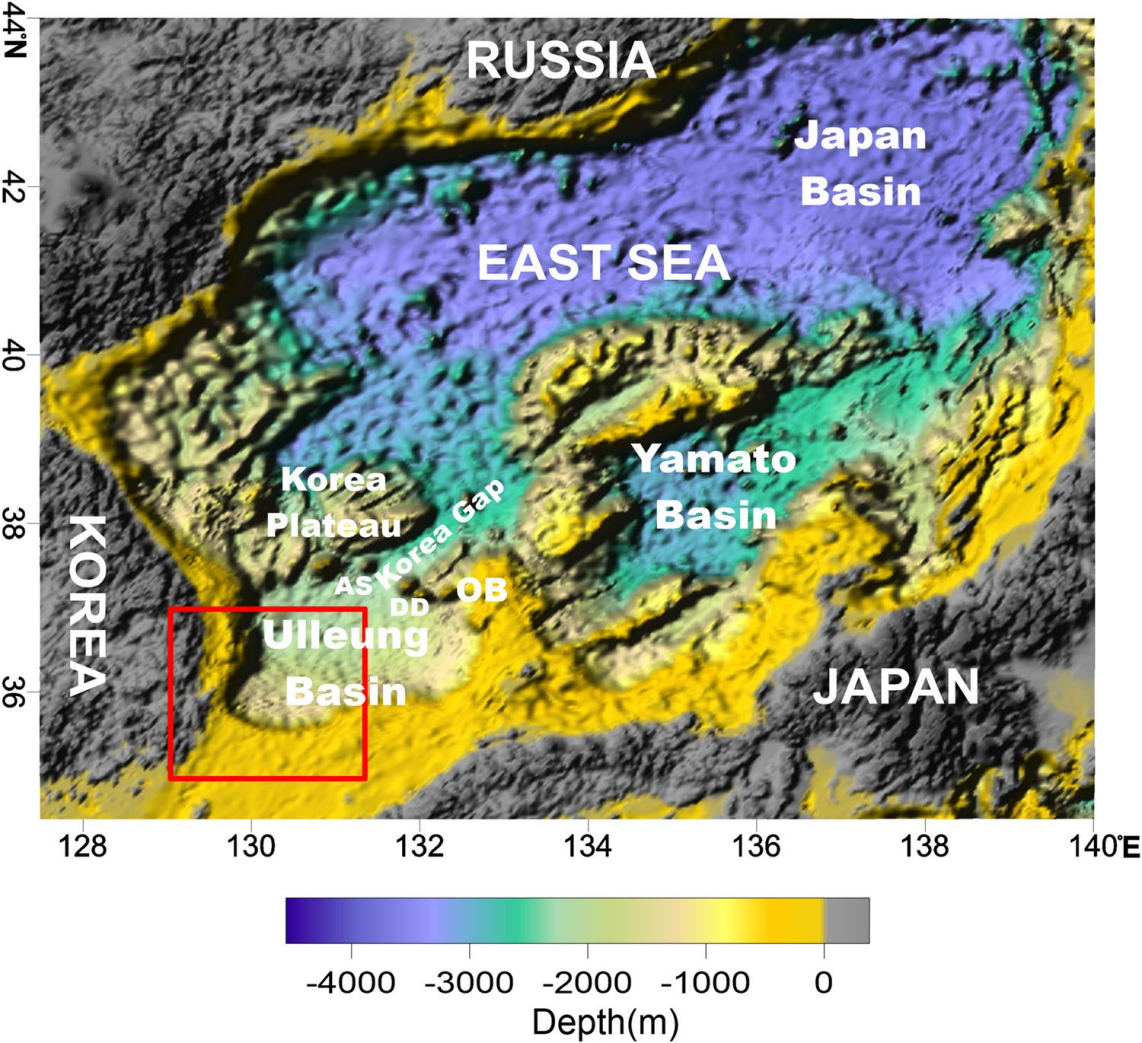
Bathymetry of the East Sea (Sea of Japan). The East Sea is surrounded by Korea, Japan, and Russia, and includes three deep basins. The red box represents the study area. AS – Anyongbok seamount; DD – Dokdo (Dok Island); OB – Oki bank. This figure was generated using the Surfer software (https://www.goldensoftware.com/).

The East Sea is a marginal sea in the western Pacific and is a typical back-arc basin that faces the circum-Pacific orogenic belt where active crustal deformation occurs. The East Sea was formed by back-arc spreading that initiated during the late Oligocene or early Miocene when the Pacific Plate began subducting beneath the Eurasian plate along the Japan Trench, and part of the eastern Eurasian continent broke off^10–12^. Most of the East Sea is more than 2000 m deep and is divided into the Ulleung, Japan, and Yamato basins (Fig. 1). These basins developed independently and are separated by ridges and plateaus, including the Yamato Ridge and the Korean Plateau. These plateaus and ridges are fragments of the continental crust that remained in the East Sea when Japan separated from the Eurasian continent^13^.

**Figure 2:**
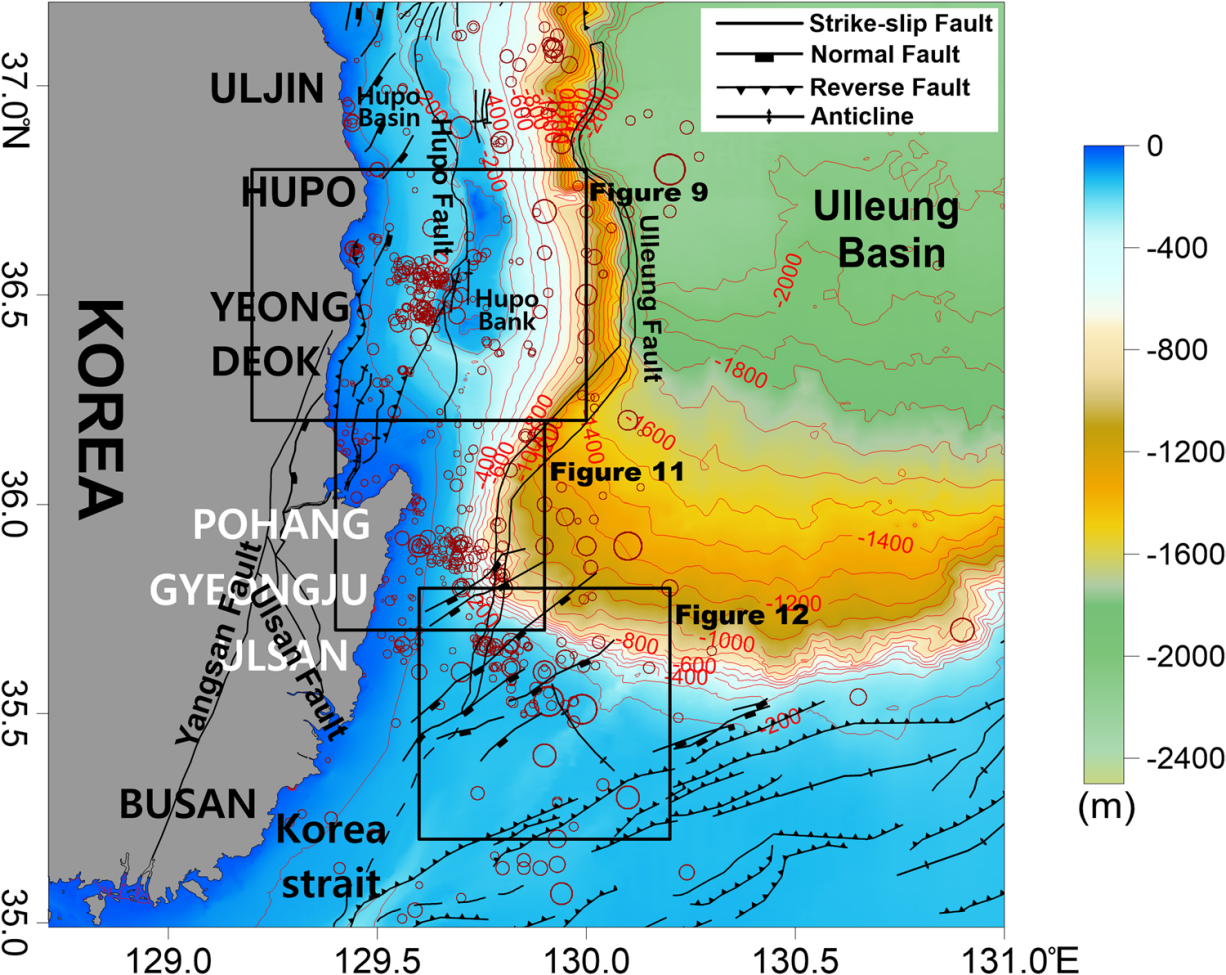
Bathymetry map of the study area with major geologic structures and earthquake locations. Bathymetric contours are shown in meters. Structural data were compiled from the reports of Yoon et al.^14^ and Son et al.^15^. The black boxes represent the map areas in Figs. 9, 11, and 12. The red circles represent the locations of earthquakes. The size of a circle is proportional to the magnitude of an earthquake, from 0.7 to 5.2. This figure was generated using the Surfer software (https://www.goldensoftware.com/).

The tectonic development of the Ulleung Basin and the surrounding area proceeded according to changes in the stress field acting on the basin^14^. From the late Oligocene to the early Miocene, the basin was mainly affected by tensile stress as it began to expand to the southwest. At the end of the middle Miocene, the tectonic regime affecting the East Sea changed from extensional to compressional^15,16^. Along the eastern coast of the Korean Peninsula, continuous right lateral shear occurred in the NNW–SSE direction and several Miocene pull-apart basins formed along the right lateral strike-slip faults^15,17,18^. Earthquakes have continued to occur on these faults along the eastern coast of the Korean Peninsula, which were originally associated with the opening of the East Sea. In particular, many submarine earthquakes have occurred in the southwestern part of the Ulleung Basin (Fig. 2). Since 5 Ma, the tectonic regime of East Asia has been controlled by E-W compression due to a decreased subduction angle of the Pacific plate and the eastward movement of the Amurian plate^19,20^. Earthquake activities in East Asia may have been affected by the E-W compressive stress. Most gravity and magnetism studies of the Ulleung Basin have focused on its regional tectonic structures^21,22^ and the gravity and magnetic properties of the marine volcanoes^23–25^. However, the gravity and magnetic characteristics of the earthquake-prone regions in the Ulleung Basin have not yet been studied.

Thus, this study aimed to analyze the gravitational and magnetic properties of the seafloor, to determine the tectonic characteristics of submarine earthquake-prone areas in the southwestern part of the Ulleung Basin. We examined free-air, isostatic gravity anomalies, magnetic anomalies, reduction to the pole (RTP) magnetic anomalies, and a tilt derivative. The earthquake-prone areas were divided into three regions for comparing and investigating the gravity and magnetic results. The results elucidate the characteristics of the tectonic structure, which will aid in understanding the processes of seismic hazards.

### Geologic setting

The East Sea formed in response to subduction of the Pacific and Philippine plates, the Japanese islands separated from the Eurasian margin^26^. The deep Ulleung, Yamato, and Japan basins in the East Sea are separated by the Oki and Kita-Yamato banks, the Yamato Ridge, and the Korea Plateau (Fig. 1)^13^.

The Ulleung Basin is located in the southwestern part of the East Sea and is separated from the Yamato and Japan basins by the Oki Bank and the Korea Plateau, respectively. The seafloor of the Ulleung Basin is fairly smooth, and deepens northeastward from ~ 1000 m at the basin margin to ~ 2300 m near the Korea Gap, located between Anyongbok seamount and Dokdo (Dok Island) (Fig. 1)^23,24,27^. The basin is bounded by the Korean Peninsula to the west, Oki Bank to the east, the Korea Plateau to the north, and the Korea Strait to the south^28^.

Studies involving ocean bottom seismometer, gravitational, magnetic, and other geophysical data have indicated that the velocity structure of the Ulleung Basin is similar to that of typical oceanic crust, although the crust is approximately twice as thick as normal oceanic crust^13,21,27,29,30^. Scientists have suggested a variety of origins for the abnormally thick oceanic crust observed in the Ulleung Basin; however, its formation is not yet clearly understood^11,12,27^.

Rotational, pull-apart, and combined structural models have been suggested to explain the origin of the East Sea^10,12,13,31,32^. The opening of the East Sea is generally thought to have begun during the late Oligocene or early Miocene, continuing until ~ 15–12 Ma. The Ulleung Basin formed during the final stage of the opening of the East Sea^27,33–35^.

The northward collision of the Izu-Bonin Arc against Honshu Island initiated the clockwise rotation of the Honshu block during the early–middle Miocene^11^. The tectonic regime along the southwestern margin of the Ulleung Basin also changed from extensional to compressional^36,37^. The compressional stress regime produced E–W shortening, accompanied by the folding and thrusting of early–middle Miocene sequences along the eastern Korean continental margin^14,15^. Many fault systems are present around the Ulleung Basin and the Korea Strait. The Hupo and Ulleung faults, with small folds and thrust faults, are located along the western margin of the Ulleung Basin (Fig. 2)^14,38^. The strike-slip Ulleung Fault extends N–S to NNE–SSW along the western boundary of the Ulleung Basin. The Hupo Fault, which extends along the Hupo Basin, is a ~ 3 km wide and ~ 140 km long normal fault. At the end of the late Miocene, contractional deformation caused the convergent strike-slip reactivation of the Hupo Fault, as well as widespread reverse faulting and folding within continental slope sequences. During this time, Hupo Bank was uplifted to form a half-graben (Hupo Basin)^14,38^.

Along the southeastern margin of the Korean Peninsula adjacent to the southwestern part of the Ulleung Basin, the Miocene sedimentary basins related to the opening of the East Sea are divided into the Pohang, Janggi, Waup, Eoil, Haseo, Jeongja, and Ulsan basins from north to south^17,39,40^. They are generally bounded by the Ulsan and Yangsan faults, which are presumed to be the strike-slip faults along which the basins formed^17,39,41,42^. Several kinematic models consider the basins in southeastern Korea to be pull-apart basins related to the NNE-striking dextral strike-slip Yangsan Fault^15,41^. During the development of the basins, a WNW–ESE to NW–SE extensional stress regime was dominant in southeastern Korea^17,43–45^.

The change from an extensional to compressional tectonic regime initiated the back-arc closing stage in the Ulleung Basin. As a result of the compressive stress, large-scale folds and thrust faults striking NE–SW developed in the southern part of the basin^15^. Tsushima Island, located at the southwestern end of the Ulleung Basin, exhibits various types of folds due to NNW–SSE compression^15,46^. The Tsushima-Goto fault system, which is a major tectonic boundary around the Korea Strait, exhibits thrust faults and folds that dominate the eastern side, while normal fault systems dominate the western side^34,41,47,48^. This region also contains the Dolgorae Thrust Belt (DTB) to the northeast of Tsushima Island^15,34,46^. Dextral strike-slip faults along the DTB have formed a NW–SE anticlinal flexure^49,50^. According to seismic and gravity surveys, the southwestern continental shelf of the Ulleung Basin contains an ~ 8–10 km thick sedimentary layer owing to a substantial supply of sediment produced by the uplift and erosion resulting from the compressional tectonic regime^28,51^.

After the creation of the East Sea, the eastward movement of the Amurian plate and the decreasing subduction angle of the Pacific plate gave rise to E-W compression in East Asia from 5 Ma^19,20^. Faults and geological structures created during the formation of the East Sea have since been subjected to continuous compressive stresses (for approximately 5 Ma), which has triggered earthquake activities^4,20^.

## Materials and methods

The regional depths of the East Sea were obtained using global bathymetry data with a resolution of 30 arc seconds (SRTM30 PLUS, Becker et al.^52^). Detailed DEM data (150 m grid resampling data) collected by the National Oceanographic Research Institute of Korea with shipboard sonar systems were used to determine the bathymetry of the southwestern part of the Ulleung Basin.

**Figure 3:**
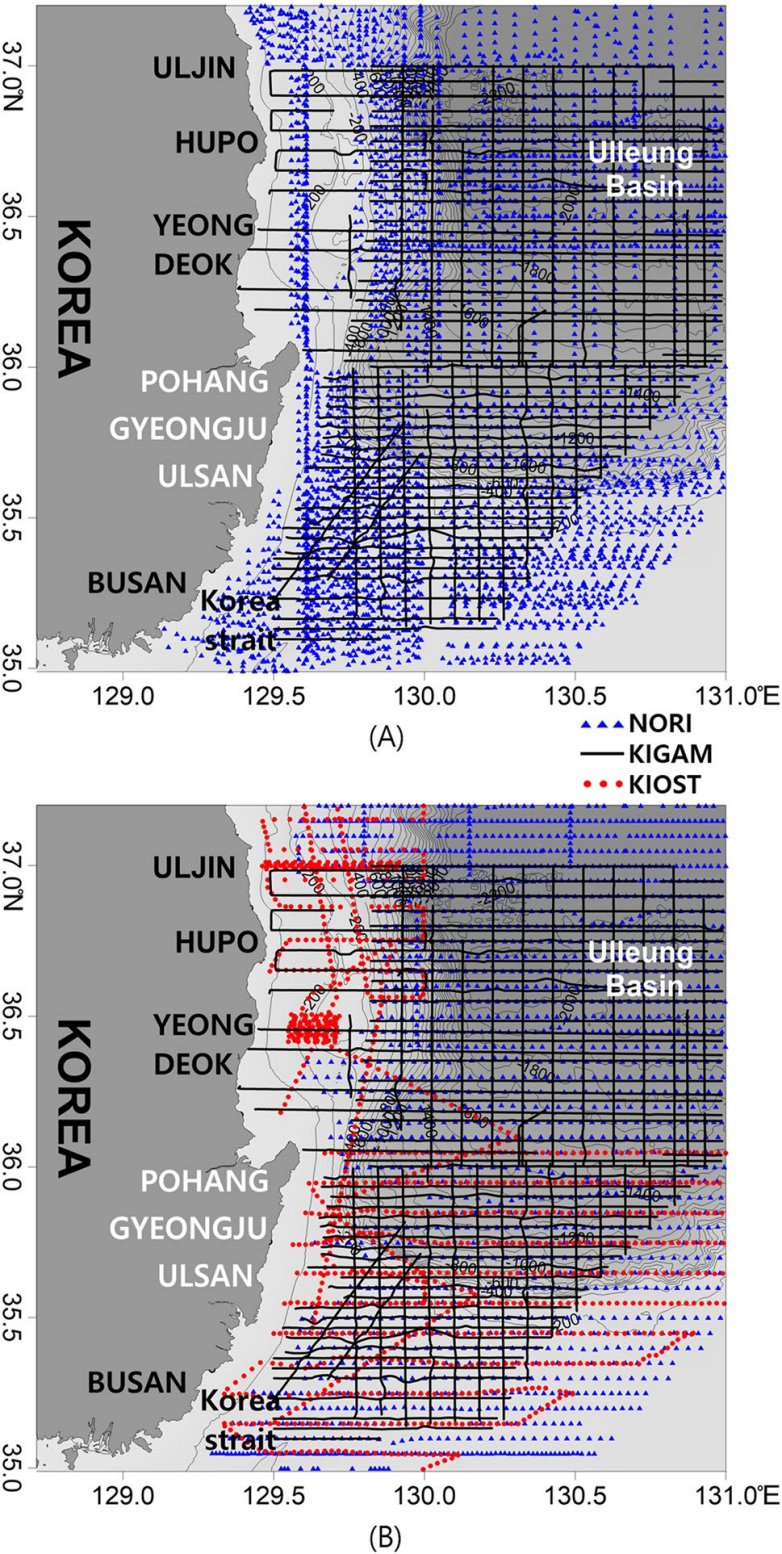
(**A**) Survey tracks for gravity data and (**B**) survey tracks for magnetic data. NORI – National Oceanographic Research Institute of Korea; KIGAM – Korea Institute of Geoscience and Mineral Resources; KIOST – Korea Institute of Ocean Science and Technology. This figure was generated using the Surfer software (https://www.goldensoftware.com/).

Gravitational and magnetic data in the study area were obtained using shipboard gravimeters (Lacoste & Romberg S-115, S-118, and MicrogLacoste MGS-6) and magnetometers (Geometrics G-811G and Marine magnetics SeaSPY). The gravity and magnetic data used in this study were collected by different institutes and during different cruises: the Korea Institute of Ocean Science & Technology (1990, 1993, 2011, 2012, and 2015 : magnetic data), the National Oceanographic Research Institute of Korea (1996 and 1997 : gravity and magnetic data), and the Korea Institute of Geoscience and Mineral Resources (2006 and 2012 : gravity and magnetic data) (Fig. 3). The survey track patterns have a spacing of 0.5–3 nautical miles. We obtained free-air gravity data and magnetic anomaly data (after IGRF (International Geomagnetic Reference Field) correction) at each institution. We encountered difficulties when combining datasets collected by different instruments at various times. When the offset between the track lines was consistent, a constant value correction was made using the average offset value^53^. We also used the Oasis Montaj stitching sub-routine in the Grid Knitting software^54^ to adjust the contrast levels of the data from the different cruises and time periods.

We analyzed the free air gravity, isostatic gravity, magnetic, and reduction to the pole (RTP) magnetic anomalies in the selected datasets. The isostatic anomaly was calculated by the Wavenumber Correlation Analysis (WCA) method using the Fourier transform with the correlation coefficient between the free air gravity anomaly and the terrain gravity effect (TGE)^55,56^. The TGE was derived by the Gauss–Legendre quadrature integral, with the densities of 2670 kg/m^3^ of crust and 1030 kg/m^3^ of seawater, according to the topographical curvature based on sea level (m.s.l). Terrain Correlated Free-air Gravity Anomaly (TCFAGA) was obtained by extracting the high correlation coefficient data of the two data sets using the WCA method, and the isostatic anomaly was derived by removing the TGE from the TCFAGA. The isostatic gravity anomaly reflects the mass distribution and equilibrium status of the crust^57,58^. RTP converts an observed anomaly into an anomaly under vertical magnetization, thereby removing any anomaly asymmetry caused by inclination and contributing to the magnetic anomaly analysis^59–61^. We used a geomagnetic inclination of 52.3° and a declination of −7.7° for the RTP transformation of the magnetic data. We then utilized the tilt derivative results of the RTP magnetic anomaly data to help constrain the shape of the magnetic body^62^. The tilt derivative method consists of the ratio of the vertical and horizontal derivatives of the RTP magnetic anomaly^63,64^. The RTP magnetic anomaly removes the inclination dependency of the tilt anomaly; thus, the zero contour of the tilt derivative is located close to the boundary of the causative magnetic body^62^.

The locations and magnitudes of submarine earthquakes that occurred from 1981 to 2019 were obtained from the Korea Meteorological Administration (https://www.weather.go.kr/w/eqk-vol/search/korea.do). The maximum earthquake magnitude measured was ~ 5.2, while the minimum was ~ 0.7.

## Results and discussion

### Bathymetry

The Ulleung Basin has a gentle slope, except around the volcanic islands and seamounts in the northeastern part of the basin (Fig. 1). The water depth in the Ulleung Basin gradually deepens from ~ 1000 m in the southwest to ~ 2300 m in the northeast where it meets the Korea Gap that connects to the Japan Basin to the north. The basin is surrounded by the steep continental slope of the Korean Peninsula to the west and by the Korea plateau to the north. In general, the slope of the seafloor in the basin is steep in the west and is relatively gentle in the south and east.

The southwestern part of the Ulleung Basin features a continental shelf connecting the steep continental slope with the deep basin to the east (Fig. 2). The southern continental shelf region in the study area is relatively flat, with a water depth of < 200 m, while the water depth changes rapidly toward the northern continental slope. The continental shelf is relatively wide in the south and gradually narrows to the northwest.

The Hupo Basin and Hupo Bank are located in the northwestern part of the study area and were formed during the opening of the East Sea. They are separated by a water depth of ~ 200 m. Hupo Bank rises almost vertically, with a flat summit at a depth of ~ 130 m or less. Hupo Bank is parallel to the eastern coastline of the Korean Peninsula and is higher than the surrounding seafloor topography. The N–S length of the bank is ~ 85 km, and the E–W width is ~ 1 km at its narrowest and ~ 16 km at its widest. The flat top of Hupo Bank has a water depth of ~ 130–140 m, similar to the regression sea level during the Quaternary Ice Age^65^. The bank is presumed to have been affected by erosion during the last glacial maximum. The water depth in the Hupo Basin west of Hupo Bank is ~ 100–250 m.

Earthquakes in the study area were mainly distributed on the continental shelf within a water depth of ~ 200 m and on the continental slope. Three earthquake-prone regions were observed: the southern part of Hupo Bank located east of Yeongdeok (~ 100–250 m depth), the Pohang offshore region (~ 50–500 m depth), and the Ulsan offshore region, which is the farthest from land (~ 150–180 m depth).

### Gravity anomalies

**Figure 4:**
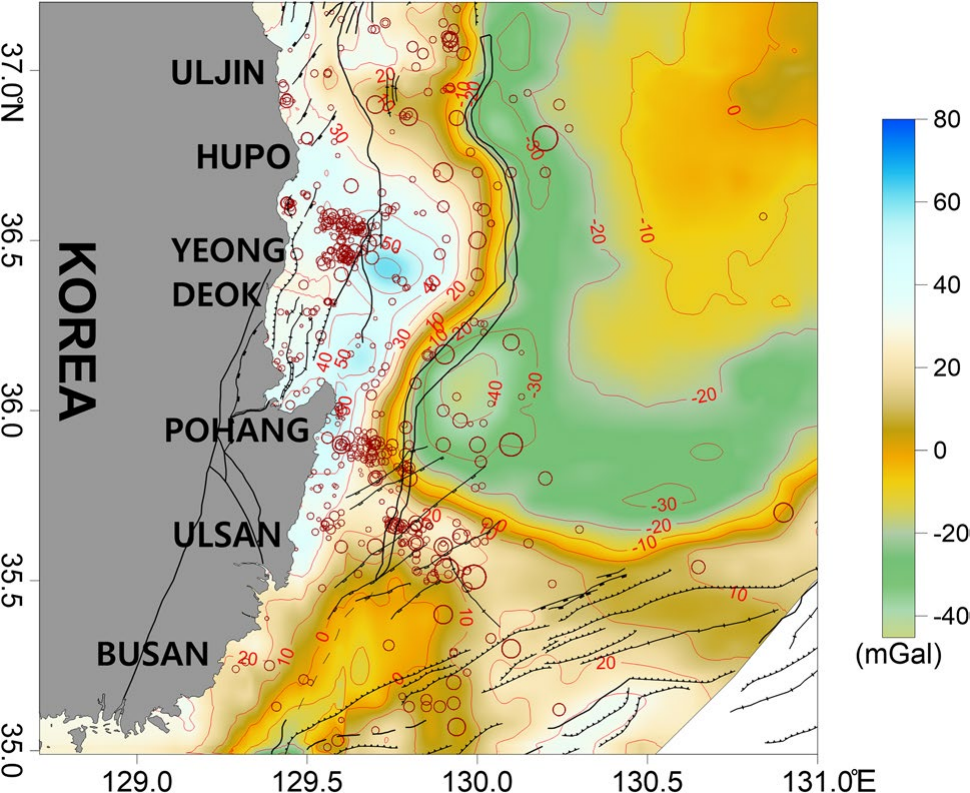
Free-air gravity anomaly map of the study area. Contour intervals are 10 mGal. The major geologic structures and symbols of the earthquakes are the same as those in Fig. 2. This figure was generated using the Surfer software (https://www.goldensoftware.com/).

Free-air gravity anomalies generally reflect the influence of terrain. The free-air gravity anomalies in the study area were mainly influenced by the seafloor bathymetry and ranged from approximately −50 to 70 mGal (Fig. 4). High anomalies were observed along the coast and continental shelf, while low anomalies were observed on the continental slope and in the Ulleung Basin. The overall distribution of the anomalies was similar to the changes in seafloor bathymetry.

In the Hupo Basin, the anomalies were lower than in the surrounding areas because of the locally depressed bedrock and the thick sedimentary layer. On Hupo Bank, a bathymetric peak adjacent to the eastern part of the basin exhibited high anomalies (Fig. 4). Considering the overall bathymetry of Hupo Bank, a high free-air gravity anomaly should extend from north to south over the entire Hupo Bank region. However, a high anomaly was only observed on the southern part of Hupo Bank. The gravity anomaly decreased sharply as the continental slope deepened to the east. Dehlinger^66^ suggested that changes in the gravity anomaly are typical crustal edge effects at the continental crust boundary. The continental slope to the west of the Ulleung Basin has been interpreted as the boundary of the continental crust^30,67^. The steep change in the gravity anomaly seen in this study supports this result. In the flat and deep Ulleung Basin at the bottom of the continental slope, the gentle anomalies ranged from ~ 10 to −40 mGal. The free-air anomaly increased slightly toward the center of the Ulleung Basin, where the water depth reaches ~ 2300 m. This is considered to be related to crustal thinning and the relatively shallower depth of the Moho in this area^21,68^.

Many earthquakes were distributed around the high anomalies observed in the southern part of Hupo Bank (Fig. 4). Earthquakes in the Pohang offshore region were also mainly located at the edge of the high anomaly zone extending from Pohang to the southern part of Hupo Bank. In the Ulsan offshore region, more earthquakes occurred in areas with weaker high anomalies than in the other areas.

**Figure 5:**
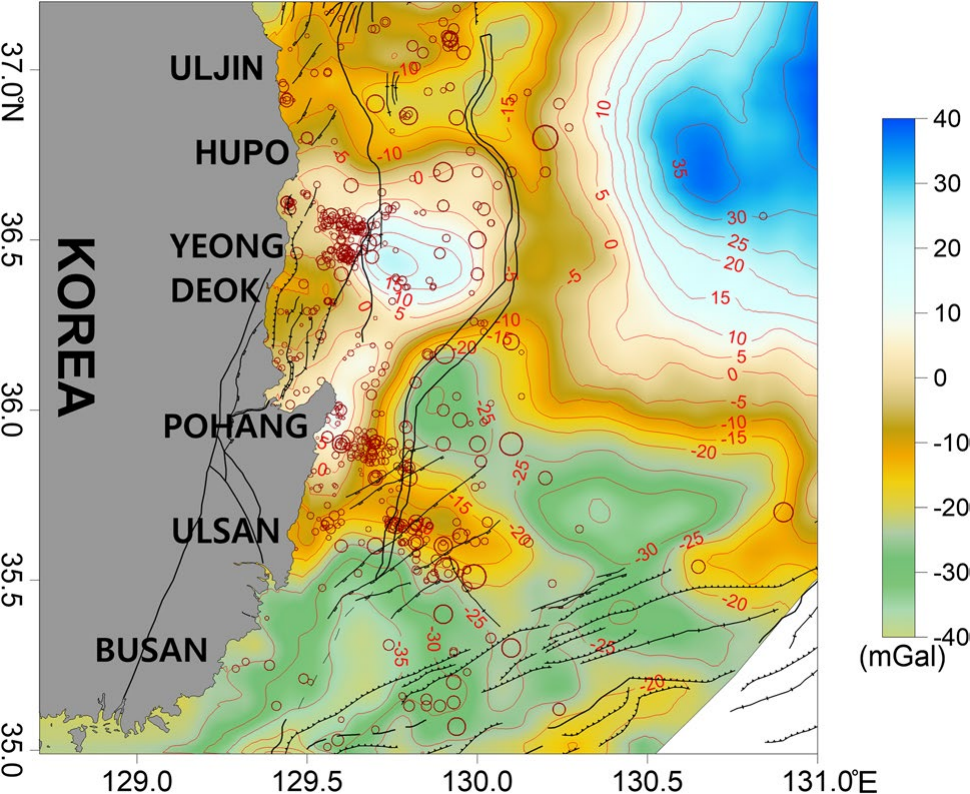
Isostasy gravity anomaly map of the study area. Contour intervals are 5 mGal. The major geologic structures and symbols of the earthquakes are the same as those in Fig. 2. This figure was generated using the Surfer software (https://www.goldensoftware.com/).

The isostatic gravity anomalies ranged from -40 to 40 mGal, and the high anomaly from Pohang to Hupo Bank exhibited the same distribution as the free-air anomaly (Fig. 5). Compared to the tectonic boundary, the high anomaly is located between the fault zone that connects the Pohang–Yeongdeok–Hupo and Ulleung fault zones. The isostatic gravity anomaly was also high at the center of the Ulleung Basin. This is likely due to rising dense mantle beneath the basin, indicating that the depth of the Moho becomes shallower towards the Ulleung Basin^21,68^. In the Ulsan offshore region, earthquake-prone areas exhibited higher anomalies than those to the south.

### Magnetic anomalies

**Figure 6:**
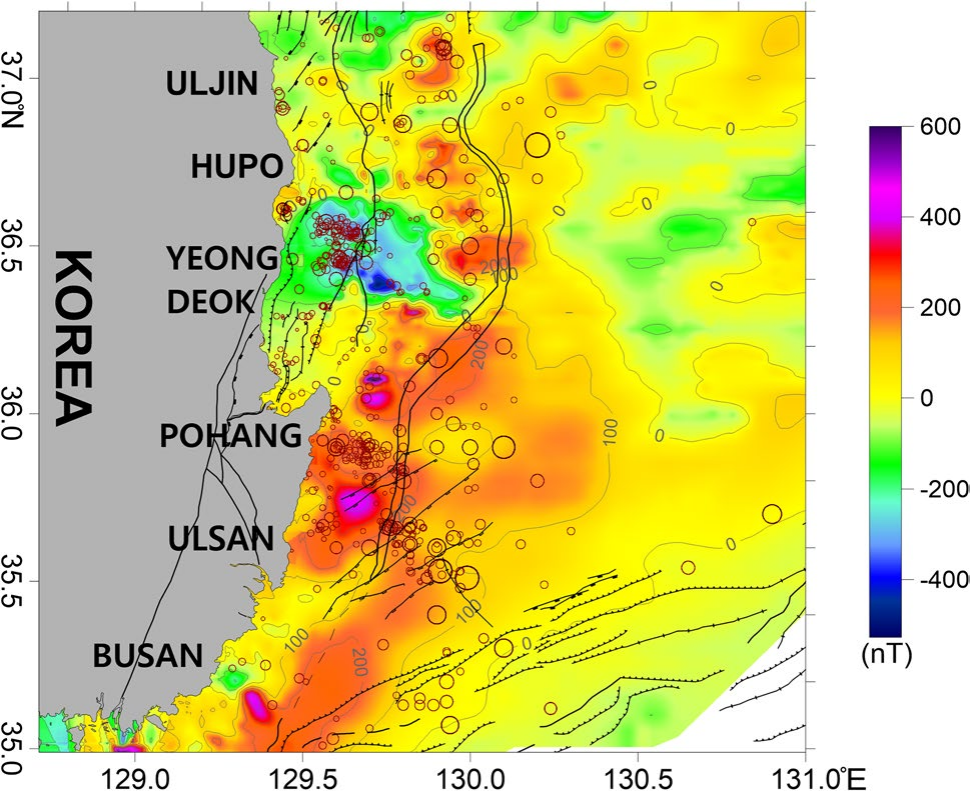
Magnetic anomaly map of the study area. The major geologic structures and symbols of the earthquakes are the same as those in Fig. 2. This figure was generated using the Surfer software (https://www.goldensoftware.com/).

Strong magnetic anomalies indicate the presence of strongly magnetized subsurface geologic structures, such as igneous rocks. Therefore, if strong magnetic anomalies are observed on seafloor areas without artificial magnetic structures or subsurface mineral resources, the presence of igneous rocks can be inferred. Magmatic activity related to the opening of the East Sea occurred along the eastern coast of the Korean Peninsula, the continental shelf, and the continental slope^14,69,70^. Strong magnetic anomalies were predominant in these areas. In the study area, highly positive magnetic anomalies were mainly distributed on the continental shelf and slope (Fig. 6). However, the Ulleung Basin, with its thick sediment layer, generally exhibited weak magnetic anomalies with limited fluctuations. In the Hupo Basin and Hupo Bank to the east of Yeongdeok, highly negative magnetic anomalies were observed, and several other negative magnetic anomalies were observed along the coast (Fig. 6). Highly positive magnetic anomalies were observed south of the negative magnetic anomalies on Hupo Bank.

**Figure 7:**
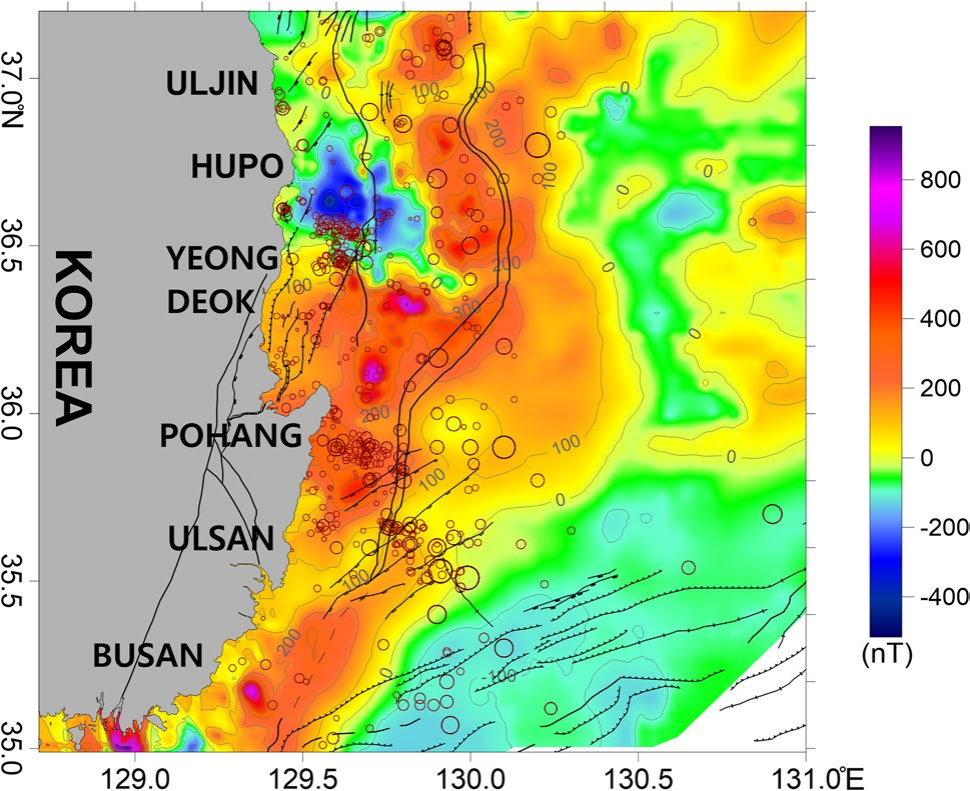
Reduction to the pole (RTP) magnetic anomaly map of the study area with a geomagnetic inclination of 52.3° and declination of −7.7°. The major geologic structures and symbols of the earthquakes are the same as those in Fig. 2. This figure was generated using the Surfer software (https://www.goldensoftware.com/).

The RTP method removes any asymmetry caused by inclination. The RTP magnetic anomalies were strong in locations where earthquakes frequently occurred (Fig. 7). Strong magnetic anomalies were also observed in the earthquake-prone areas east of Yeongdeok and Pohang. These regions experienced earlier magmatic activity related to the opening of the East Sea, suggesting that they were affected by magma rising along cracks in the crust^14,30^. In addition, volcanic deposits such as basalt and tuff in land near the coast support the occurrence of magmatic activity^15,18,71,72^. The RTP magnetic anomaly map shows magnetic dipole anomalies on Hupo Bank and in the Hupo Basin (Fig. 7). The anomalous zones indicate possible prior magmatism in the area. In addition, the eastern continental slope near Hupo Bank and Pohang exhibited strong magnetic anomalies, which we interpreted as strongly magnetized seaward-dipping reflectors (SDRs)^30^. The strong magnetic anomalies in the southwestern area of the Ulleung Basin reflect the presence of intrusive or extrusive igneous rocks, which formed as a result of large-scale volcanism along the tectonic boundary during the rifting and expansion of the East Sea. This area has also been interpreted as the transition region from continental crust to oceanic crust^30,67^. In contrast, the earthquake-prone Ulsan offshore region did not exhibit strong magnetic anomalies compared to the other regions.

**Figure 8:**
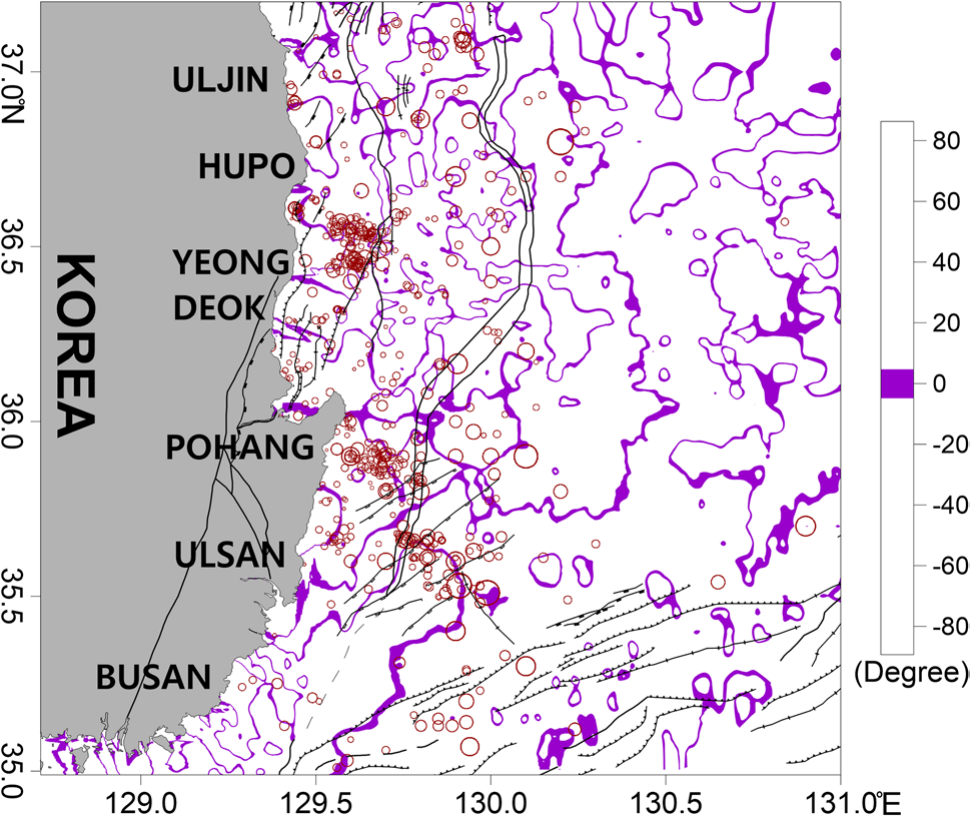
Tilt derivative map of the RTP magnetic anomaly of the study area. Thick purple lines represent contours between 5 and −5°, indicating the boundaries of magnetic source bodies. The major geologic structures and symbols of the earthquakes are the same as those in Fig. 2. This figure was generated using the Surfer software (https://www.goldensoftware.com/).

The tilt derivative of the RTP magnetic anomaly was calculated to estimate the magnetic structures in the crust. The zero contour of the tilt derivative is located near the boundaries of magnetic bodies. The tilt derivative map exhibits contours between 5 and −5°, centered around 0° (Fig. 8). Except for those in the deep Ulleung Basin, earthquakes in the study area were mainly located around the zero contour of the tilt derivative. Therefore, we assumed that the earthquakes occurred more frequently near the boundaries of the magnetic structures. Thus, the tilt derivative method might be useful for comparing the locations of earthquakes and geologic structures.

### Southern area of Hupo bank

**Figure 9:**
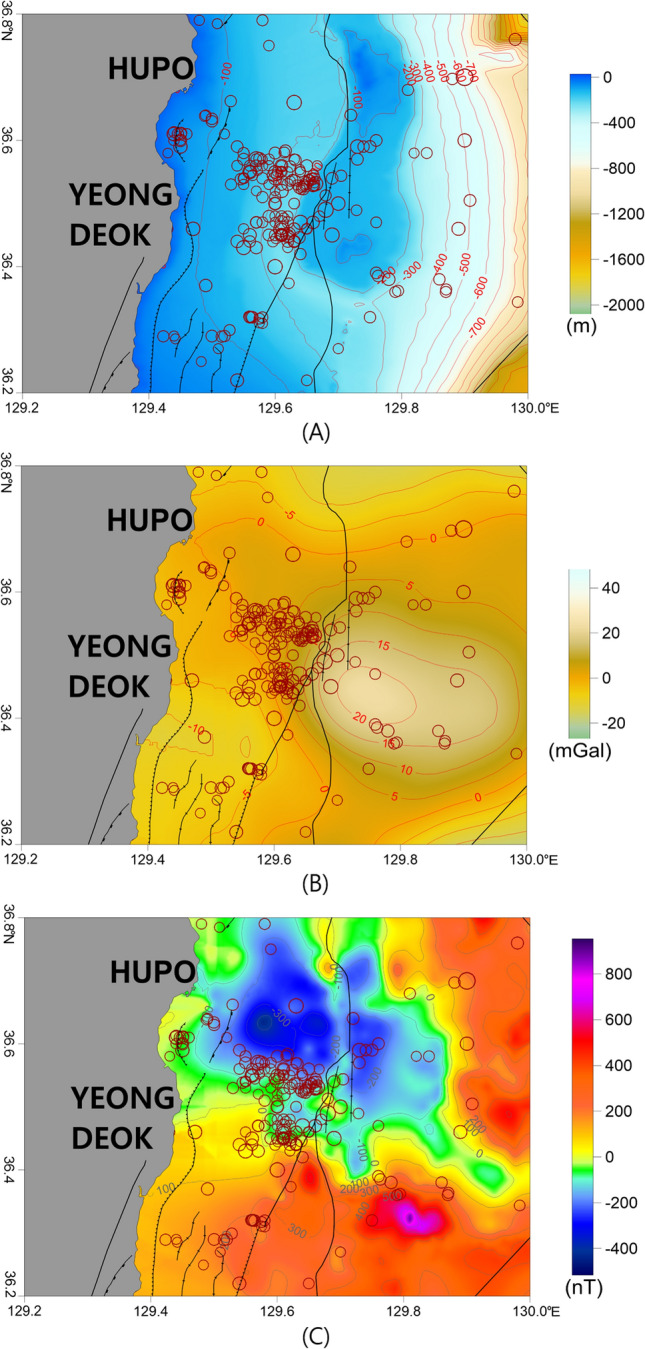
(**A**) Bathymetry map, (**B**) isostasy gravity anomaly map, and (**C**) RTP magnetic anomaly map of the southern area of Hupo Bank. These major geologic structures and symbols of the earthquakes are the same as those in Fig. 2. The figures were generated using the surfer Software (https://www.goldensoftware.com/).

In the earthquake-prone area of Hupo Bank, 138 earthquakes occurred between 1982 and 2019 with magnitudes ranging from 0.7 to 3.6. Comparing with the reference point of the Gyeongju earthquake (Sep. 12, 2016), 86 and 52 earthquakes occurred before and after the earthquake, respectively. The earthquakes were concentrated in the southern part of Hupo Bank and the Hupo Basin (Figs. 2 and 9). Earthquakes were not only observed on Hupo Bank in the east, but also in the Hupo Basin in the west.

Hupo Bank is considered to be a massif that broke off the eastern coast of the Korean Peninsula when the East Sea formed, and contains rock formations similar to those found along the eastern coast^41,73^. The entire Hupo Bank region, which is a long elevated section of rocky seafloor with a summit that extends from north to south, experienced tectonic motion related to the formation of the Hupo Basin^41,74^. Based on the overall bathymetry of the Hupo Bank area, a high gravity anomaly should be observed over the entire Hupo Bank. However, only the southern region of the bank exhibited a high gravity anomaly (Figs. 4, 5, and 9), and earthquakes have also concentrated around the southern region. These results indicate the presence of gravity effects based on differences in geologic structures, rather than simple topographic effects.

The isostastic gravity anomaly on the southern part of Hupo Bank exhibited a clear connection with the high anomaly in the northern part of Pohang (Fig. 5). These connected zones are located between the two tectonic boundaries of the Pohang–Yeongdeok–Hupo and Ulleung fault zones. Based on the elongated high gravity anomaly that extends from Pohang to the southern part of Hupo Bank, we inferred that the connection area was heavily influenced by dense igneous rocks related to the opening of the East Sea^14,41,67,74^, or that the geological structures may differ from those in other regions.

In the earthquake-prone region, a strong dipole anomaly was observed in the RTP magnetic anomaly data, with a large range of −400–700 nT (Figs. 7 and 9). These strong magnetic anomalies likely indicate the influence of strong magmatic activity. In the southern parts of Hupo Bank and the Hupo Basin, the tilt derivative of the RTP magnetic anomaly indicates that the earthquakes were predominantly located near zero contours associated with the boundaries of the magnetic structures (Fig. 8). However, the seafloor topography in this area did not have distinct volcanic features (Figs. 2 and 9). In this case, the strong magnetic anomalies in the Hupo Basin and Hupo Bank might indicate magmatic activity below the seafloor during the opening of the Hupo Basin. Because many earthquakes were concentrated along the Hupo Fault Zone of Hupo Bank, as well as in the Hupo Basin, we inferred that the basin contains crustal fractures under the thick sedimentary layer^14,74^, although these fractures are not expressed on the seafloor. In addition, strong magnetic anomalies within the basin suggest the influence of magmatic activity related to the fractures and faults below the basin.

**Figure 10:**
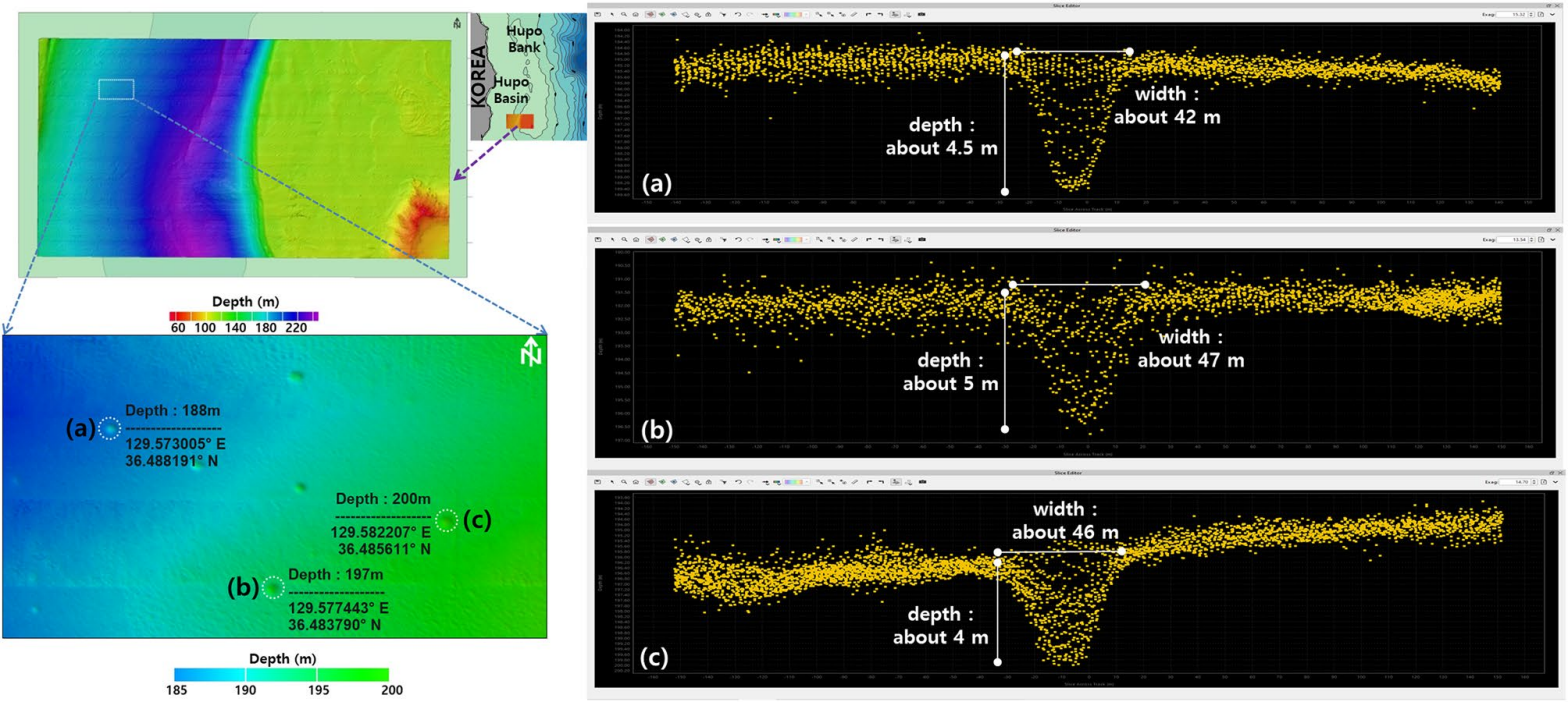
Bathymetry and pockmarks in the southern part of the Hupo Basin (Left) and various sizes of pockmarks in the survey area (Right). These figures were generated using the Surfer software (https://www.goldensoftware.com/), the Hips & Sips software (http://www.teledynecaris.com/), and the Fledermaus software (http://www.qps.nl/).

In 2011, the Korea Institute of Ocean Science & Technology (KIOST) conducted a detailed bathymetric survey using a multi-beam echosounder in parts of the southern Hupo Basin and Hupo Bank^75^. Pockmarks, which are crater-like structures recessed into the seafloor^76–78^, were observed in the survey area of the Hupo Basin (Fig. 10). In general, pockmarks are used as evidence and indicators of current and past fluid activity in the seafloor and indicate concentrated fluid flow. Although pockmarks have several possible origins, studies have shown that they can result from groundwater pressure and fluid flow related to earthquakes^79–82^. Pockmarks occur mostly in fine-grained surface sediments with low permeability^76–78^. Sediments collected from the pockmark region of the Hupo Basin were mainly composed of fine-grained silt, satisfying this condition. The pockmarks were mainly distributed at depths of 150–250 m in the Hupo Basin (Fig. 10)^75^. The pockmarks identified in the survey area had diameters of approximately 20–50 m and depths of approximately 4–6 m. Hundreds of pockmarks were observed in the survey area in the southern Hupo Basin. It is likely that additional pockmarks would be observed if the survey area was expanded to include the entire Hupo Basin. As mentioned above, the southern part of the Hupo Basin and Hupo Bank contains faults^14,74^ and is an earthquake-prone area. Therefore, the pockmarks may indicate changes in subsurface fluid pressure associated with these earthquakes.

### Pohang offshore region

**Figure 11:**
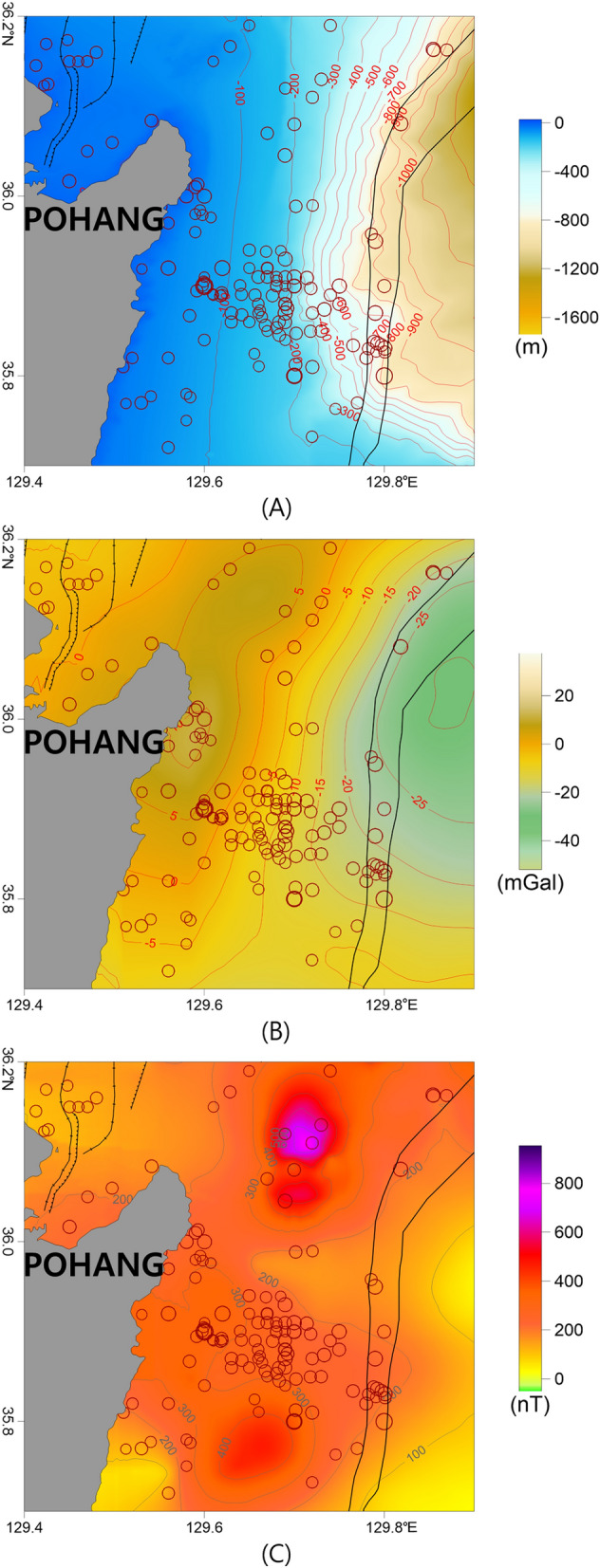
(**A**) Bathymetry map, (**B**) isostasy gravity anomaly map, and (**C**) RTP magnetic anomaly map of the Pohang offshore region. The major geologic structures and symbols of the earthquakes are the same as those in Fig. 2. These figures were generated using the Surfer software (https://www.goldensoftware.com/).

In the Pohang offshore region, the water depth increased eastward (Figs. 2 and 11). In this region, earthquakes occurred from the coast below a depth of ~ 100 m to the continental shelf and slope. Between 1981 and 2019, ~ 95 earthquakes occurred in this area, with magnitudes ranging from ~ 0.9 to 3.8. The numbers of earthquakes before and after the Gyeongju earthquake were 46 and 49, respectively.

A high gravity anomaly has been observed in the land of Pohang, which is related to magmatic activity during the formation of the East Sea^15,18,71,72^. We presumed that there is a relationship between the terrestrial gravity anomaly and the marine gravity anomaly, which extends from Pohang to the southern part of Hupo Bank (Figs. 4, 5, and 11). The earthquake-prone region east of Pohang is located on the east side of the extended high gravity anomaly. Except for the Ulleung Fault to the east, no specific tectonic boundaries were observed on the coast or the continental shelf in this area^14,70,74^. The water depths of this region gradually increase seaward from the continental shelf. The presence of the high gravity anomaly, without any specific bathymetric changes in the earthquake-prone area, suggests that there may be rocks of different densities beneath the seafloor or that the tectonic structure may be different.

Positive RTP anomalies were mainly distributed in the earthquake-prone region and the continental shelf and slope west of the Ulleung Fault exhibited strong positive magnetic anomalies (Figs. 7 and 11). The tilt derivative map indicates that the earthquakes were largely located near zero contours related to the magnetic structure boundaries (Fig. 8). We presumed that magmatic activity related to the opening of the East Sea occurred in this area, as in the Hupo Basin and Hupo Bank. This magmatism has also been interpreted as being related to terrestrial igneous rocks of the Tertiary Janggi Group in Pohang^15,18,40,72^. In addition, volcanic rocks such as basalt and tuff are widely distributed in the coastal regions of the southeastern Korean Peninsula, including the Tertiary Janggi Group^15,40,72^. K–Ar dating revealed that these volcanic rocks have ages of 25–17 Ma^71^. Therefore, these volcanic rocks are assumed to be closely related to the rifting of the eastern coast of the Korean Peninsula and the formation of the East Sea, including the Ulleung Basin. Furthermore, it is inferred that the above-mentioned gravity anomalies of Pohang-Hupo Bank connected from Pohang land, and the high magnetic anomalies are closely related to the high gravity zones and terrestrial igneous activities in the land area of the east coast^15,18,71,72^. Kim et al. ^30^ suggested that the continental slope contains many SDRs that are coincident with magnetic anomalies and are related to the rifting of the southwestern margin of the East Sea. The strong magnetic anomalies observed on the continental slope, east of Youngdeok and Pohang, are therefore inferred to be the result of these SDRs. This continental slope area is likely the boundary of the continental crust, which would be accompanied by magmatic activity^14,30,67^. The high gravity anomalies and strong magnetic anomalies in these regions are likely related to the distribution of dense igneous rocks due to subseafloor magmatism. These results indicate that crustal fissures, where magmatic activity and earthquakes could occur, might be located around the anomalies.

### Ulsan offshore region

**Figure 12:**
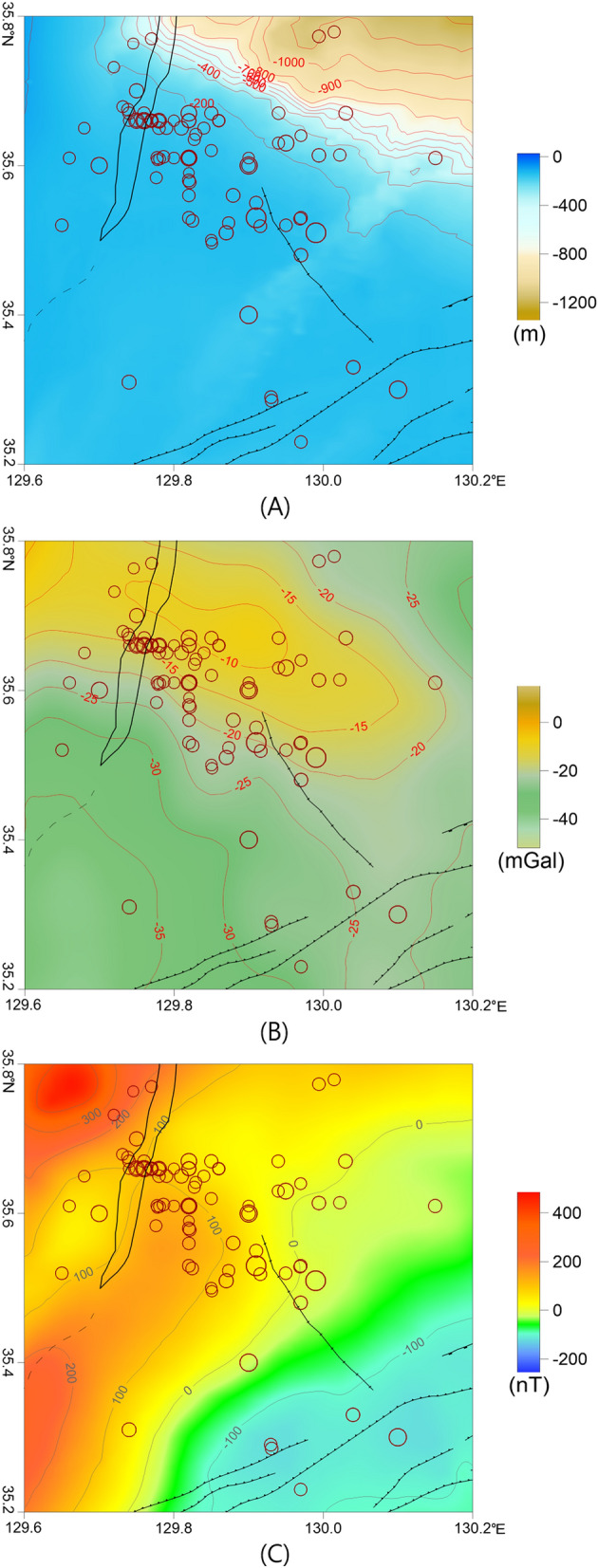
(**A**) Bathymetry map, (**B**) isostasy gravity anomaly map, and (**C**) RTP magnetic anomaly map of the Ulsan offshore region. The major geologic structures and symbols of the earthquakes are the same as those in Fig. 2. These figures were generated using the Surfer software (https://www.goldensoftware.com/).

The Ulsan offshore region contained many earthquakes that were farther from land than those in the other two areas. Approximately 69 earthquakes occurred from 1995 to 2019, with magnitudes ranging from ~ 1.0 to 5.0 (Figs. 2 and 12). In this area, 56 and 13 earthquakes occurred before and after the Gyeongju earthquake, respectively. This region has a wide continental shelf that extends to the Korea Strait in the southwest and is connected to the deep Ulleung Basin via the continental slope in the northeast (Fig. 2). The water deepens rapidly to the north; however, there is little change in water depth to the south. The earthquakes in this area occurred mainly in a NW–SE direction within the continental shelf.

Although the gravity anomaly observed in this area was weaker than that of the Pohang–Yeongdeok–Hupo anomaly, it was linearly oriented in a NW–SE direction (Figs. 4, 5, and 12). The earthquake sites were mostly located at the southern edge of this anomaly. The gravity anomaly was located in an area with little variation in seafloor bathymetry, compared to the southern part of the anomaly. Therefore, the contrasting parts of this anomaly are presumed to be caused by the differences in the subseafloor structure.

The area has a weaker RTP magnetic anomaly than those of the other two earthquake-prone regions, with a range of ~ 100–0 nT (Figs. 7 and 12). The gentle magnetic anomaly suggests that the potential for magmatic activity is lower in this region than in Hupo or Pohang. However, the tilt derivative of the RTP magnetic anomaly in this area indicates that the earthquakes were mainly located near zero contours related to the magnetic source boundaries (Fig. 8).

Among the marginal areas of the Ulleung Basin, this area is known to contain a very thick sedimentary layer^14,51,83–85^. The eastern and western sides of the Tsushima-Goto tectonic line around the Korea Strait in the southwestern part of the Ulleung Basin have different geological structures^15,34,46,48^. On the eastern side of the Tsushima-Goto Line, the DTB and Gorae structures were produced by compressive stress^14,34,46,86^. Lee et al.^87^ reported that low-velocity seismic layers are widespread in this region across a range of depths. The low velocity zone was supposedly influenced by the thrust fault zone or mantle upwelling. Kim et al.^46^ and Son et al.^15^ suggested that several normal faults are located on the western side of the Tsushima-Goto Line. Considering the weak magnetic anomaly and the linear gravity anomaly observed here, the earthquake-prone region in the Ulsan offshore region is likely due to differences in the underlying geologic structures, rather than the effect of magmatic activity. The southwestern part of the Ulleung Basin underwent sequential tensional and compressional tectonic regimes during the formation of the East Sea, which likely resulted in the complex tectonic zone observed today^15,34,46,48^. Earthquakes in the Ulsan offshore region are therefore considered to result from complex geologic structures.

## Summary and conclusions

Three earthquake-prone areas, namely the southern part of Hupo Bank, the Pohang offshore region, and the Ulsan offshore region, were identified in our study area within the Ulleung Basin. The gravitational and magnetic characteristics of the geological structures in these areas were analyzed and the following conclusions were drawn.

The southern parts of Hupo Bank exhibited high gravity anomalies and strong magnetic dipole anomalies with ranges of −10–25 mGal for the isostatic gravity anomaly and −400–700 nT for the RTP magnetic anomaly. These anomalies are interpreted as representing the effects of igneous activity related to the opening of the East Sea. In addition, pockmarks observed in the Hupo Basin suggest that earthquake activity may be an indicator of subsurface fluid pressure changes.

The Pohang offshore region exhibited a high gravity anomaly, with a range of −25–10 mGal, that extended from Pohang to Hupo Bank, as well as a strong magnetic anomaly with a large range of 200 to 700 nT. Similar to those in the Hupo region, these anomalies likely resulted from magmatic activity related to the opening of the Ulleung Basin. Earthquakes in the above two areas are likely the result of movements of unstable fracture zones due to the influences of past magmatic activities and related geological structure differences.

In the Ulsan offshore region, a linear gravity anomaly was observed. This area exhibited a weaker magnetic anomaly than the other two regions. This area is influenced by complicated geological structures, owing to the alternating tensional and compressional tectonic regimes. Earthquakes are presumed to have occurred frequently along these complex structures, including thrust faults, anticlinal folds, and normal faults.

After the formation of the East Sea, it has been under E-W compressive stress from 5 Ma. Previous studies^20,88,89^ suggest that recent earthquakes in the Korean Peninsula and the offshore area of Yeongdeok and Ulsan were also influenced by the compressive stress. Therefore, it was assumed that the unstable tectonic structures associated with the formation of the East Sea, including the Ulleung Basin, have been affected by this stress, resulting in the possibility of earthquakes. In the southwestern part of the Ulleung Basin, submarine earthquakes around Gyeongju (Hupo Bank, Pohang offshore) became more frequent after the Gyeongju earthquake. Before the earthquake in Gyeongju, the southern part of Hupo Bank and the Pohang offshore had averages of 2.5 earthquakes and 1.3 earthquakes per year, respectively. However, since then, these rates increased to averages of 17.3 earthquakes and 16.3 earthquakes per year, respectively. The number of earthquake observations can be increased regarding more sensitive measurement of small earthquakes due to the development of equipment observation technology. However, the impact of Gyeongju earthquakes cannot be excluded. Therefore, earthquake monitoring should be performed in the seafloor regions with gravity and magnetic anomalies, containing subsurface tectonic structures that influence seismicity.

## Data Availability

The datasets used during the current study are available from the corresponding author on reasonable request.
